# The action of different irrigant activation methods on engineered endodontic biofilm: an *in vitro* study

**DOI:** 10.2340/biid.v12.43065

**Published:** 2025-04-11

**Authors:** Aya Awaida, Roula El Hachem, Aline Issa, Mireille Kallasy, Carla Zogheib, Wajih Hage

**Affiliations:** a Department of Endodontics, Faculty of Dentistry, Saint Joseph University of Beirut, Beirut, Lebanon; b Craniofacial Research Laboratory, Division of Biomaterials, School of Dentistry, Saint Joseph University of Beirut, Beirut, Lebanon; c Faculty of Public Health, Lebanese University, Fanar, Lebanon; d Faculty of Pharmacy, Saint Joseph University, Beirut, Lebanon; e Saint Joseph University of Beirut, Life Sciences, Campus of Sciences and Technologies, Beirut, Lebanon

**Keywords:** Activation techniques, bacterial count reduction, Eddy, endodontic infections, EndoUltra, irriflex, irrigation, multispecies biofilm, XP Finisher

## Abstract

**Introduction:**

Endodontic infections are biofilm-mediated, demanding effective biofilm eradication from the root canal. Root canal complexities, coupled with bacterial biofilm resistance, pose challenges to thorough disinfection. Irrigation, particularly with sodium hypochlorite, is crucial in endodontics. Activation techniques, like sonic or ultrasonic oscillations, enhance irrigant penetration and biofilm disruption, improving decontamination and treatment outcomes.

The aim of the present study was to evaluate the effectiveness of XP Finisher, EndoUltra, Eddy and Irriflex in the reduction of the multispecies endodontic biofilm formed by *Enterococcus faecalis, Pseudomonas aeruginosa, Candida albicans and Proteus mirabilis.*

**Methods:**

A total of 44 single-rooted mandibular premolars were selected and divided into groups for investigation: Group A: Irriflex, Group B: XP Finisher, Group C: Eddy system, and Group D: EndoUltra system. Multispecies biofilms, comprising *Enterococcus faecalis, Proteus mirabilis, Pseudomonas aeruginosa,* and *Candida albicans*, were cultured and inoculated into the pre-treated dentinal canals, which were then incubated for 16 days. Following this, the canals were subjected to the respective irrigation protocols. Bacterial counts were assessed using sterile paper points and culture techniques post-irrigation. Additionally, four non-inoculated root canals were used as negative controls for comparison.

**Results:**

EndoUltra achieved the highest reduction in Total Bacterial Count (TBC) with a median decrease of 75% (interquartile range [IQR]: 70–80%), significantly better than XP Finisher (*p* = 0.001) and Irriflex (*p* = 0.001). Eddy led to a reduction in *Pseudomonas aeruginosa* (PA) with a median decrease of 85% (IQR: 80–90%), significantly outperforming Irriflex (*p* = 0.001) and XP Finisher (*p* = 0.001). For *Enterococcus faecalis* (EF), EndoUltra had a median reduction of 70% (IQR: 65–75%), significantly better than Eddy (*p* = 0.01) and Irriflex (*p* = 0.001), while XP Finisher resulted in a reduction of 60% (IQR: 55–65%). EndoUltra showed the highest reduction in *Proteus mirabilis* (ProM) with 80% (IQR: 75–85%), significantly better than Irriflex (*p* = 0.001) and XP Finisher (*p* = 0.001), with Eddy also better than Irriflex (*p* = 0.009). EndoUltra reduced *Candida albicans* (CA) by 65% (IQR: 60–70%), significantly outperforming XP Finisher (*p* = 0.001) and Eddy (*p* = 0.001).

**Conclusion:**

Within its limitations, this study identified EndoUltra as highly effective in reducing bacterial counts, indicating its potential utility in disinfecting root canals. These findings underscore the significance of such methods in enhancing treatment outcomes and addressing root canal infections.

## Introduction

Endodontic infections, characterised by polymicrobial consortia residing within the intricate network of the root canal system, present a formidable challenge in achieving successful root canal therapy outcomes [1]. Central to the resilience of these infections is the formation of endodontic biofilms, which constitute a complex and organised community of microorganisms embedded within an extracellular polymeric substance [2]. This biofilm matrix serves as a protective fortress, rendering the microorganisms highly resistant to conventional disinfection agents [3]. The persistence of this biofilm can lead to treatment failure, underscoring the pressing need for effective irrigation techniques in root canal therapy.

To address the tenacity of endodontic biofilms, various activation techniques have been employed in conjunction with irrigants, with the aim of augmenting their antimicrobial efficacy [4]. Among the most widely utilised irrigants, sodium hypochlorite (NaOCl) stands as a cornerstone due to its remarkable antimicrobial properties [5]. When subjected to mechanical activation through techniques such as sonic, ultrasonic, or alternative modalities, NaOCl can effectively infiltrate and disrupt the biofilm architecture, enabling more comprehensive disinfection [6].

The activation of irrigants in endodontics holds paramount importance in achieving effective disinfection within the root canal system. This process serves as a critical adjunct to traditional irrigation methods, as it addresses the limitations posed by stagnant, passive delivery of irrigants. Activation techniques, whether through mechanical means such as sonic or ultrasonic oscillations, impart dynamic energy to the irrigant, thereby enhancing its penetration into complex anatomical irregularities and biofilm matrices [7]. This mechanical agitation promotes the disruption of bacterial colonies and facilitates the removal of organic and inorganic debris, ultimately leading to a more thorough decontamination of the root canal space [8]. Furthermore, activation techniques have been shown to enhance the reach of irrigants into lateral canals and isthmuses, which are notoriously challenging to access through conventional means [6]. By harnessing the power of activation, we aim to elevate the standard of endodontic disinfection and thereby improve the likelihood of successful treatment outcomes.

This study examined the efficacy of four distinct activation techniques: Irriflex system (Produits Dentaires SA, Vevey, Switzerland), XP Finisher (FKG Dentaire, La Chaux-des-Fonds, Switzerland), Eddy (VDW, Munich, Germany) (sonic irrigation), and EndoUltra (Vista Dental Products, United States) (ultrasonic activation) in comparison with the conventional irrigation protocol. The Irriflex system, a novel development in endodontic irrigation, employs a unique dual-chamber syringe design which enables precise control and simultaneous delivery of irrigants and suction [9]. The XP Finisher, utilising a proprietary design, facilitates enhanced flow dynamics and targeted irrigation delivery within the root canal system [10]. Eddy, employing sonic activation, harnesses acoustic streaming and cavitation effects to augment the penetration and disruption of biofilm structures [7]. EndoUltra, an ultrasonic activation technique, employs high-frequency oscillations to enhance the irrigants’ reach and mechanical disruption within the root canal system [8].

The aim of this study was to ascertain the superior activation technique among the Irriflex system, XP Finisher, Eddy (sonic irrigation), and EndoUltra (ultrasonic activation) in reducing the multispecies endodontic engineered biofilm.

## Material and methods

The study protocol was approved by the “Ethics Committee” of Saint Joseph University of Beirut, Lebanon (Study Reference: FMD Tfemd/2024/2). The study was conducted in the Microbiological Laboratory at the same university.

### Specimen selection and preparation

A total of 44 single-rooted mandibular premolars were chosen for this investigation, divided into four groups of 10 teeth each, with an additional 4 serving as negative controls. Selected premolars exhibited intact crowns without prior restorations. Preliminary radiographs, captured from both mesio-distal and bucco-lingual perspectives, verified the existence of a solitary root canal. Premolars exhibiting fractured or immature apices were omitted from the study.

The included premolars underwent external root surface scaling and were immersed in a 0.9% saline solution at 4°C for 24 h. Following this, the samples were taken out of the solution and rinsed with distilled water.

After creating the access cavity, root canal patency was established using a 10 K-flexofile (Dentsply, Maillefer, Ballaigues, Switzerland) until it became visible through the apical foramen. The working length was set at 1 mm shorter than the apex. With this information, the roots were further trimmed to achieve a final working length of 16 mm. Root canals were subsequently enlarged in accordance with the manufacturer’s guidelines, sequentially up to a size of 25/0.05 RegularShape file from ProgressShape (Rekita, Lebanon). Alternatively, irrigation was carried out using 5.25% NaOCl (Clorox, Vernon, CA, USA) throughout the shaping process, employing a 3 mL syringe and a 27G lateral side-ject in a back and forth movement of 2mm amplitude needle (Transcodent GmbH & Co., Sulzer, Switzerland) at a rate of 0.05 mL/s. Following the final NaOCl irrigation, 3 mL of distilled water was used to remove any residual solution. Finally, a last flush of 17% EDTA (Vista, WI, USA) was employed as a concluding irrigation (3 mL, 0.05 mL/s for 1 minute). Each root canal was thoroughly dried with sterile paper points.

To prevent bacterial leakage, the foramen was sealed with resin-epoxy material, and the teeth were embedded in blocks of silicone impression material (3M ESPE Express STD, MN, USA).

Subsequently, the specimens underwent sterilisation for 20 minutes at 121°C under 20 PSI pressure using a W&H-Lisa autoclave (Bürmoos, Austria).

### Formation of multispecies biofilm

*Enterococcus faecalis*, sourced from ATCC 29212 and cultivated aerobically on blood agar at 35°C for 48 h following manufacturer’s guidelines, was subsequently grown in Brain Heart Infusion + 5% glucose (BHI) broth at 37°C for 24 h in a shaker incubator, followed by a further 24-h static incubation at 37°C. An inoculum was then prepared in sterile BHI + 5% glucose broth, with turbidity adjusted to 0.5 McFarland, equivalent to approximately 1.5 × 108 colony forming units per millilitre (CFU/mL).

Ten microlitres of this inoculum were applied onto 44 dentinal canals pre-treated with collagen type 1 and incubated for 16 d at 37°C.

*Proteus mirabilis* (ATCC 12453) and *Pseudomonas aeruginosa* (ATCC 27853) were cultured on Plate Count Agar (PCA) at 37°C for 24 h following manufacturer’s instructions. *Candida albicans* (ATCC 10231) was cultured on Yeast Glucose Chloramphenicole (YGC). Colonies were then cultured in BHI + 5% glucose broth at 37°C for 24 h in a shaker incubator, followed by an additional 24-h static incubation at 37°C. An inoculum was prepared in sterile BHI + 5% glucose broth, with turbidity adjusted to 0.5 McFarland, equivalent to approximately 1.5 × 108 CFU/mL.

*Pseudomonas aeruginosa* was introduced to the pre-treated dentinal canals on day 10, *Candida Albicans* on day 14, and *Proteus mirabilis* on day 16. Following the addition of all microorganisms, the multispecies biofilm was incubated for an additional 10 d at 37°C.

After the incubation period, canal roots were divided into 4 groups:

Group A (*n* = 10): The Irriflex application involved a gentle vertical movement of 3 mm, positioned 1 mm from the apical terminus of the preparation. This distinctive needle, facilitated the controlled delivery of a 3 mL solution of NaOCl at 5.25% concentration, administered over a 5-minute period, equating to a flow rate of 0.05 mL/s. This method served as the experimental group for the irrigation protocol.

Group B (*n* = 10): The XP Finisher was placed in a contra-angle handpiece (VDW Silver). It was then removed from the plastic tube with a lateral movement in rotation mode. Each canal received 0.5 mL of 5.25% NaOCl, after which the instrument was inserted without rotation. Rotation was initiated at 800 rpm and 1 Ncm, and the instrument was activated for 1 minute with slow and gentle 7–8 mm lengthwise movements up to the WL. Subsequently, the XP-endo Finisher tool was withdrawn from the canal, and a final irrigation protocol was performed using 4.5 mL of 5.25% NaOCl with the syringe/needle positioned 1 mm short of the WL. Each instrument was used in two teeth before being discarded.

Group C (*n* = 10): The sonically activated irrigation with the Eddy system (VDW, Munich, Germany) involved using a flexible polyamide tip sized 25.04, which was driven by an air hand piece (AirScaler-W&H, Lisa, Bürmoos, Austria). The tip was moved up and down over a distance of 3 mm, starting 1 mm from the apical terminus, without applying pressure, following the manufacturer’s guidelines (operating at 6000 Hz, with an intensity of 0.3 mPa/3 bar) for a duration of 30 s. This procedure was repeated three times. After each activation cycle, the canal was flushed with 3 mL of irrigant (administered at a rate of 0.05 mL/s) using Irriflex, which contained 5.25% NaOCl.

Group D (*n* = 10): Ultrasonic irrigation was conducted using the EndoUltra system (MicroMega, Besancon, France). This cordless device employs a 15/0.2 activator tip that oscillates at a frequency of 40 KHz. The procedure involved the use of 5 mL of 5.25% NaOCl solution, with the activator tip moved vertically over a distance of 3 mm, applying no pressure, for a duration of 30 s, starting 1 mm from the apical terminus, in accordance with the manufacturer’s instructions. This process was repeated three times. After each activation cycle, the canal was flushed with 3 mL of 5.25% NaOCl using a 27G needle.

After irrigation and drying of the root canals, the bacterial count was assessed. This involved inserting a sterile paper point into each canal for 5 minute. Subsequently, the paper points were transferred to 500 µL of sterile BHI broth for 15 minute. After vortex mixing, 50 µL of the liquid medium was serially diluted in sterile BHI broth and plated on various agars. PCA was used to determine the total bacterial count (TBC), YGC for *Candida albicans*, Cetrimid Agar (AC) for *Pseudomonas aeruginosa*, Slantez and Bartley Agar (SBA) for *Enterococcus faecalis*, and Uriselect agar for *Proteus mirabilis*. The culture media were then incubated at 37 °C for 48 h. Colony counting and confirmation were conducted by observing colony morphology on blood agar and performing Gram staining.

Four non-inoculated root canals were also incubated and cultured to act as negative controls (*n* = 4).

### Statistical analysis

Data were analysed using the Statistical Package Software for Social Science (SPSS for Windows, Version 23.0, Chicago, IL, USA). Descriptive statistics of the quantitative variables (difference in unit count between T1 and T0) for each group and for each category (TBC, *Pseudomonas aeruginosa*, *Enterococcus faecalis*, *Proteus mirabilis*, *Candida albicans*) were summarised and presented as medians (interquartile range [IQR]) and means ± standard deviations (SDs).

The normality of the distribution of the quantitative variables was assessed using the Shapiro-Wilk test and by visually inspecting the histogram shape when the Shapiro-Wilk test yielded inconclusive results (*p*-value between 0.45 and 0.05). When data were not normally distributed or equality of variances was not assumed, the Kruskal-Wallis test was used to compare values between the four groups. Post hoc pairwise comparisons of groups were performed, with *p*-values adjusted using the Bonferroni correction to account for multiple comparisons.

Two *p*-values are presented in Table 2 for each comparison: ‘Sig.’ represents the unadjusted significance value, while ‘Adj. Sig.’ refers to the Bonferroni-adjusted significance value. This ensures that the statistical inference accounts for the increased likelihood of Type I errors due to the multiple pairwise comparisons.

To compare the efficacy of each technique on unit counts, the difference between the first count and the last count (‘Diff’) was computed for each category and technique. The univariate analysis of count reduction for each category across the four groups of comparison is summarised in Table 1. All tests were two-tailed, and the level of significance was set at 5%.

**Table 1:** Univariate analysis of the count reduction T1–T0 (x10^4^) per species for each of the four activation techniques included in the study (*N* = 40).

Categories	Irriflex (*n* = 10)	XP Finisher (*n* = 10)	Eddy (*n* = 10)	EndoUltra (*n* = 10)
**Total Bacterial Count**				
*Mean ± SD*	-1,878,900 ± 101.5928	-1,698,250 ± 0.08475	-499918.7 ± 0.00856	-1,498,530 ± 0.04522
*Median (IQR)*	-1,882,500 (1.85000)	-1,698,650 (0.11750)	-1,499,978 (0.01490)	-1,498,700 (0.09250)
*Shapiro-Wilk p-value*	0.106	0.005	0.004	0.032
***Pseudomonas aeruginosa***				
*Mean ± SD*	-157,860 ± 0.07321	-148,270 ± 0.06976	-118,340 ± 0.03238	-138,480 ± 0.03994
*Median (IQR)*	-157,700 (0.1175)	-148,550 (0.0850)	-118,450 (0.0475)	-138,550 (0.0750)
*Shapiro-Wilk p-value*	0.480	0.011	0.185	0.050
***Proteus mirabilis***				
*Mean ± SD*	-176,980 ± 0.0891	-166,380 ± 0.0532	-148,520 ± 0.0311	-148,350 ± 0.0313
*Median (IQR)*	-176,800 (0.1125)	-166,600 (0.0850)	-148,650 (0.0325)	-148,300 (0.0575)
*Shapiro-Wilk p-value*	0.516	0.091	0.003	0.105
***Enterococcus faecalis***				
*Mean ± SD*	-167,860 ± 0.0951	-139,660 ± 0.0087	-149,805 ± 0.0042	-129,839 ± 0.0056
*Median (IQR)*	-168,100 (0.1200)	-139,660 (0.0202)	-149,795 (0.0072)	-129,845 (0.0080)
*Shapiro-Wilk p-value*	0.081	0.179	0.419	0.045
***Candida albicans***				
*Mean ± SD*	-117,780 ± 0.0597	-119941.1 ± 0.0047	-129,832 ± 0.0049	-109,815 ± 0. 0038
*Median (IQR)*	-117,800 (0.0950)	-119968.5 (0.0089)	-129,830 (0.0077)	-109,810 (0.0060)
*Shapiro-Wilk p-value*	0.996	0.015	0.426	0.479

SD: Standard Deviation; IQR: Interquartile range Q3-Q1; GRP1: Conventional irrigation using IRRIFLEX; GRP2: XP FINISHER; GRP3: EDDY; GRP4: ENDOULTRA.

**Table 2:** Bivariate analysis showing statistical significance in reducing *Enterococcus faecalis* count between the four techniques.

Sample 1-Sample 2	Test statistic	Std. error	Std. Test statistic	Sig	Adj. Sig.
Conventional irrigation using Irriflex-EDDY	-10.000	5.225	-1.914	0.056	0.334
Conventional irrigation using Irriflex-XP Finisher	-20.000	5.225	-3.827	0.000	0.001
Conventional irrigation using Irriflex-ENDOULTRA	-30.000	5.225	-5.741	0.000	0.000
EDDY-XP Finisher	10.000	5.225	1.914	0.056	0.334
EDDY-ENDOULTRA	-20.000	5.225	-3.827	0.000	0.001
XP Finisher-ENDOULTRA	-10.000	5.225	-1.914	0.056	0.334

Each row tests the null hypothesis that the Sample 1 and Sample 2 distributions are the same.

Asymptotic significances (2-sided tests) are displayed. The significance level is.05.

‘Sig.’ represents the unadjusted significance value, while ‘Adj. Sig.’ refers to the Bonferroni-adjusted significance value.

## Results

### TBC reduction

When comparing the different techniques in reducing the TBC, EndoUltra induced the highest reduction in TBC followed by Eddy and XP Finisher. The conventional irrigation technique Irriflex showing the lowest impact on TBC reduction (fure 1).

Bivariate analysis showed a highly significant difference between EndoUltra and XP Finisher (*p* = 0.001) and between EndoUltra and Irriflex (*p* = 0.001) respectively, EndoUltra inducing the highest reduction in TBC. However, no statistical difference was found between EndoUltra and Eddy regarding TBC reduction.

Moreover, Eddy was significantly superior to Irriflex (*p* = 0.001) in reducing TBC. No statistical difference was found between Eddy and EndoUltra in terms of TBC reduction.

### Pseudomonas aeruginosa count reduction

The Eddy technique induced the highest reduction in PA count followed by EndoUltra and XP Finisher, Irriflex technique having the lowest impact on PA count reduction (Figure 2).

**Figure 1:**
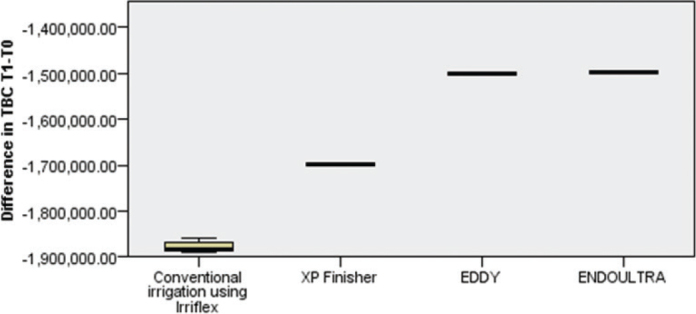
Difference in total bacterial count reduction between the four activation techniques.

**Figure 2:**
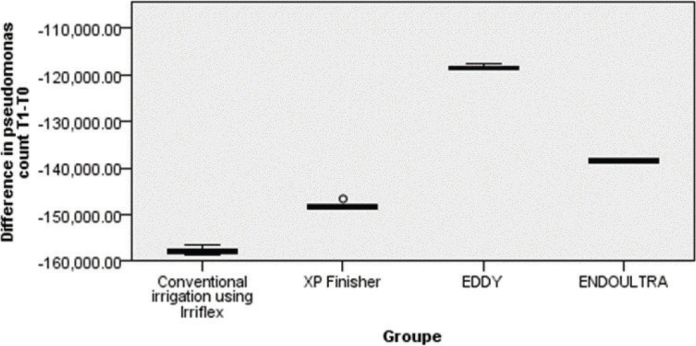
. *Pseudomonas aeruginosa* count reduction comparison between the four techniques.

Bivariate analysis showed a highly significant difference in reducing the PA count between Eddy and Irriflex (*p* = 0.001), also between Eddy and XP Finisher (*p* = 0.001), the Eddy technique showing the highest effect in reducing PA count.

Also, the EndoUltra. technique showed a significantly higher reduction in PA count compared to Irriflex (*p* = 0.001).

Moreover, no significant difference could be demonstrated between Irriflex and XP Finisher, XP Finisher and EndoUltra nor between EndoUltra and Eddy.

### Enterococcus faecalis count reduction

The EndoUltra technique induced the highest reduction in EF count followed by XP Finisher and Eddy with Irriflex having the lowest reduction in EF count.

Bivariate analysis was conducted to check the significance of this difference in bacterial count reduction and showed a highly significant difference between EndoUltra and Eddy (*p* = 0.01) and between EndoUltra and Irriflex (*p* = 0.001) with EndoUltra having the highest effect in reducing bacterial count.

Also, the XP Finisher technique showed a significantly higher reduction in bacterial count compared to Irriflex (*p* = 0.001).

**Figure 3:**
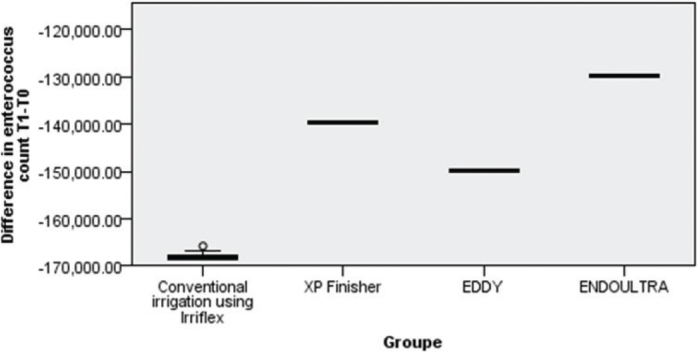
. Comparative results in reducing *Enterococcus faecalis* count between the four techniques.

No statistical difference was demonstrated between Irriflex and Eddy, Eddy and XP Finisher, XP Finisher and EndoUltra in terms of EF count reduction (Figure 3 and Table 2).

### Proteus mirabilis count reduction

The EndoUltra technique induced the highest reduction in ProM count followed by Eddy and XP Finisher. Conventional method Irriflex had the lowest impact on ProM count reduction.

A highly significant difference in reducing bacterial count (*p* = 0.001) was found between EndoUltra and Irriflex, between EndoUltra and XP Finisher as well (*p* = 0.001), with the EndoUltra technique showing the highest effect in reducing ProM count.

Also, the Eddy technique showed a statistically significant higher reduction in bacterial count compared to Irriflex (*p* = 0.009).

Although the bacterial count reduction was superior with XP Finisher compared to Irriflex, the difference was not significant between the two methods.

No statistical difference in bacterial count reduction was detected between Eddy and XP Finisher nor between EndoUltra and Eddy.

### Candida albicans count reduction

We also studied the difference in CA reduction between the four techniques and descriptive results showed that EndoUltra induced the highest reduction in CA count followed by Irriflex and XP Finisher with the EDDY technique having the lowest impact on CA count. Bivariate analysis showed a highly significant difference in reducing CA count (*p* = 0.001) between EndoUltra and XP Finisher as well as between EndoUltra and EDDY (*p* = 0.001) and with EndoUltra showing the highest reduction in CA count.

Moreover, the Irriflex technique showed a significantly higher reduction in CA count compared to EDDY (*p* = 0.001).

Although XP Finisher showed better results in reducing CA counts compared to EDDY, the difference was not statistically significant.

Conventional Irriflex technique was not statistically better than XP Finisher in reducing CA count neither was EndoUltra compared to Irriflex.

## Discussion

Endodontic pathology focusses mostly on biofilm-mediated infections, especially those brought on by surface-dwelling bacteria. The elimination of bacterial biofilm from the root canal system is the main goal of treatment for these illnesses. For understanding the pathogenic potential of root canal microbiota and creating novel disinfection techniques, it is essential to grasp the biofilm idea in endodontic microbiology. In addition to providing protection from the host’s immune system, the biofilm that root canal bacteria produce increases their resistance to several disinfectants used in irrigation methods, infection treatments, and root canal therapy [11].

Biofilm removal and good biofilm bacteria killing are required for successful therapy of many disorders.

The root canal system, on the other hand, has an extremely complicated anatomy, with isthmuses, lateral extensions, apical deltas, lateral canals, and dentinal tubules providing protection for microorganisms from instruments and disinfectants [12].

The challenge is made even more difficult by the bacteria’s biofilm lifestyle in the root canal. The microbial cells stick to the canal walls after being submerged in an extracellular matrix that they generate on their own. Compared to their planktonic relatives, biofilm cells are significantly more resistant to most antibiotics and host defences [13].

The main objectives of an endodontic treatment are to preserve the surrounding periodontal tissues and to shape and clean a root canal system. The irrigation system is a crucial component of a root canal treatment, even if its mechanical components receive most of the focus [14].

All over the years, many materials have been used to clean the root canal of a tooth, and certainly, NaOCl is the gold standard in endodontics [15].

Putting NaOCl inside of a canal is mostly done using a normal syringe, but many techniques have been involved in this process, including the use of XP Finisher, EndoUltra, Eddy and Irriflex to increase the efficacy of irrigant solutions, and especially of NaOCl [16].

The aim of the present study was to evaluate the effectiveness of XP Finisher, EndoUltra, Eddy and Irriflex in the reduction of the multispecies endodontic biofilm formed by *Enterococcus faecalis, Pseudomonas aeruginosa, Candida albicans* and *Proteus mirabilis.*

In this study, we tried to simulate the clinical reality as closely as possible. *Enterococcus faecalis* is the most often utilised test organism in endodontic biofilm model systems. This species has often been identified from teeth that have had root canal therapy but still have persistent apical pathosis. It is no longer thought that *E. faecalis* is the main pathogenic species in root canals. Because it grows quickly and is not at all irritable, it was frequently utilised in scientific settings. Its high isolation frequency and frequent usage as a test species in endodontic biofilm models are likely explained by these traits [17]. Unlike other research on single-species biofilms, this one adopts a novel strategy by examining the effects of irrigant agitation techniques on a multispecies biofilm. Accurate replication of real-world clinical settings is the goal of this study, which acknowledges their complexity. The objective of this work is to offer important insights into the efficacy of various agitation strategies by utilising a multispecies biofilm that replicates the microbial diversity in teeth with persistent apical pathosis that have undergone root canal therapy. This novel methodology provides a more thorough knowledge of how different agitation techniques affect biofilm removal and disinfection. It also emphasises the significance of comprehending interactions and behaviours among distinct microorganisms within a biofilm community [18].

One of the most popular biofilm model systems is the one used in the present study [19].

Our results indicated that all activation techniques used in this study significantly lowered the TBC. In fact, an essential part of endodontic therapy is the agitation technique, which includes activating the irrigating fluid inside the root canal system mechanically. In endodontics, it provides a number of significant advantages [20]. First of all, it helps to effectively remove debris, which improves the root canal system’s general cleanliness. By disturbing biofilms and enabling deeper penetration into dentinal tubules, agitation also enhances the antibacterial activity of irrigating solutions, leading to improved disinfection. Furthermore, it makes the smear layer easier to remove, exposing dentinal tubules to better intracanal medication penetration. Irrigants are more able to penetrate difficult anatomical regions when agitation methods like sonic or ultrasonic activation are used [21].

When comparing the different techniques in reducing the TBC, the EndoUltra induced the highest reduction in TBC followed by Eddy and XP Finisher. The conventional irrigation technique Irriflex showed the lowest impact on TBC reduction. Also, EndoUltra induced the highest reduction in *Proteus mirabilis*, *Enterococcus faecalis*, and *Candida albicans*. Many studies have shown that ultrasonic activation techniques are essential to irrigant efficacy [22, 23]. They work by using an endodontic file or stainless steel wire to transmit sonic energy via the irrigant [24]. The irrigant moves dynamically and extensively throughout the canal system as a result of cavitation and microstreaming caused by the dissipation of acoustic energy through the irrigant [25, 26]. Cavitation bubbles are created by acoustic waves; the energy produced when the bubble explodes is transferred to the walls of the root canal, removing the debris. The debris is subsequently removed from the canal by microstreaming, which transports it coronally [27]. Strong electrical activity along the active instrument, resulting from node production along activated files, has been proposed as the explanation for the successful action of passive ultrasonic irrigation (PUI) [26]. When the file meets the canal wall, there are several nodes along the instrument that prevent the reduction of auditory streaming. Nonetheless, there is disagreement over the function of cavitation in vivo, even if microstreaming is a biophysical force that is closely linked to endodontic files [28]. One may argue that the ultrasonic activation method’s strongest activity depends on the interaction of cavitation and acoustic streaming.

Also, the Eddy technique showed a statistically significant better reduction in bacterial count compared to Irriflex and XP Finisher. It induced the highest reduction in *Pseudomonas aeruginosa* count. It is thought that using ultrasonic rather than sonic activation produces a more velocious fluid stream, making the latter look more effective [29]. High-speed photography revealed the Eddy moving in a circular pattern with the instrument exhibiting an additional extra movement of the most apical region of the tip, similar to the movement of an elephant’s trunk, despite the fact that sonic activation is generally characterised by a one node pattern [30].

Regarding XP Finisher, it reduced also the TBC but not as much as EndoUltra and Eddy. Its metallurgy is responsible for the XP-endo Finisher file removal in the debris and smear layer removal. The NiTi alloy’s shape-memory properties are essential to the creation and production of XP-endo Finisher files. When the file cools to its martensitic phase, it becomes straight. The file’s shape memory causes it to change to the austenitic phase when it is exposed to body warmth (the canal). The manufacturer claims that the rotating mode’s austenitic-phase form enables the file to touch and clean regions that would otherwise be challenging to access with conventional tools. The file may be manually reshaped into its original straight shape once it has cooled [31].

Irriflex demonstrated the least impact on the removal of bacteria and the TBC compared to other activation techniques. In comparison to the other activation methods, Irriflex exhibited lower efficacy in reducing bacterial presence, suggesting a less effective bacterial removal outcome in this study. This finding is in agreement with several previous studies, which indicate the high importance of activation techniques [32]. Furthermore, it is noteworthy that the Irriflex propylene tip allows the irrigant solution to reach and clean the apical area, even in anatomically complex areas, due to the flexibility and 30G needle shape Although, the literature claims that there is a need to use other protocols and instruments to activate the irrigation solution and obtain adequate disinfection and cleaning of the entire endodontic space [33].

This study had several limitations which should be considered when evaluating results. First and foremost, the study was conducted *in vitro* and on teeth without anatomical complexities. However, this study cannot be conducted in vivo because the biofilm varies from one tooth to another, introducing bias into our study. Also, only four species was used in our biofilm, whereas in reality, there can be many more species in an endodontic biofilm.Furthermore, shaping of the root canals was performed before injecting the biofilm. However, during the shaping process, debris occludes the dentinal tubules. Shaping was necessary because there were several undercuts in the coronal portion of the canals, and without shaping, the biofilm would not reach the apex. Finally, the anaerobic strains living in the hostile conditions of the canal system may not have been subjected to the same clinical conditions during our laboratory work so laboratory conditions may not have fully simulated clinical conditions. This issue implies a limitation of our study regarding the counting of persistent bacterial strains after the application of different techniques. However, under nearly uniform working conditions, this can no longer be considered a study bias.

## Conclusion

In conclusion, this study delves into the intricate realm of endodontic infections, particularly the resilience of polymicrobial biofilms within root canal systems. Through a meticulous examination of four distinct activation techniques, Irriflex, XP Finisher, Eddy, and EndoUltra, our research sheds light on their varying efficacy in reducing a multispecies endodontic engineered biofilm. Notably, EndoUltra exhibited the highest bacterial count reduction, emphasising its potential as a valuable tool in root canal disinfection. The findings underscore the importance of activation techniques in enhancing treatment outcomes and refining endodontic protocols, paving the way for improved strategies in combating biofilm-mediated infections. This study contributes valuable insights to the evolving landscape of endodontic research and treatment modalities.
